# Resource-efficient quantum correlation measurements via multicopy neural network methods

**DOI:** 10.1038/s41598-025-24607-2

**Published:** 2025-11-19

**Authors:** Patrycja Tulewicz, Karol Bartkiewicz, Adam Miranowicz, Franco Nori

**Affiliations:** 1 Institute of Spintronics and Quantum Information, Faculty of Physics and Astronomy, Adam Mickiewicz University, Poznań, 61-614 Poland; 2 QSLab: Quantum Software Laboratory, Department of Engineering and Architecture (DIA), University of Parma, Parma, 43124 Italy; 3 Theoretical Quantum Physics Laboratory, Cluster for Pioneering Research, RIKEN, Wakoshi, Saitama 351-0198 Japan; 4 Center for Quantum Computing, RIKEN, Wakoshi, Saitama 351-0198 Japan; 5 Department of Physics, University of Michigan, Ann Arbor, MI 48109-1040 USA

**Keywords:** Physics, Quantum physics, Quantum information, Quantum simulation, Qubits

## Abstract

Measuring complex properties in quantum systems, such as measures of quantum entanglement and Bell nonlocality, is inherently challenging. Traditional methods, like quantum state tomography (QST), require a full reconstruction of the density matrix for a given system and demand resources that scale exponentially with system size. We propose an alternative strategy that reduces the required information by combining multicopy measurements with artificial neural networks (ANNs), resulting in a 67% reduction in measurement requirements compared to QST. We have successfully measured two-qubit quantum correlations of Bell states subjected to a depolarizing channel (resulting in Werner states) and an amplitude-damping channel (leading to Horodecki states) using the multicopy approach on real quantum hardware. Our experiments, conducted with transmon qubits on IBMQ quantum processors, quantified the violation of Bell’s inequality and the negativity of two-qubit entangled states. We compared these results with those obtained from the standard QST approach and applied a maximum likelihood method to mitigate noise. We trained ANNs to estimate degrees of entanglement and nonlocality measures using optimized sets of projections identified through Shapley’s (SHAP) analysis for the Werner and Horodecki states. The ANN output, based on this reduced set of projections, aligns well with expected values and enhances noise robustness. This approach simplifies and improves the error robustness of multicopy measurements, eliminating the need for complex nonlinear equation analysis. It represents a significant advancement in AI-assisted quantum measurements, making the practical implementation on current quantum hardware more feasible. The experimental results demonstrate improved noise robustness on the current noisy intermediate-scale quantum (NISQ) hardware, representing a practical advance in resource-efficient characterization of quantum correlations.

## Introduction

Quantum entanglement^1–3^ and Bell’s nonlocality, here referred to as the degree of Clauser–Horne–Shimony– Holt (CHSH) inequality violation^4–6^, are the bedrocks of quantum engineering, quantum information processing^7^, quantum communications (e.g., quantum teleportation)^8^, and quantum cryptography (e.g., quantum key distribution)^9^. Quantum nonlocality has become the foundation for numerous information processing protocols implemented within quantum systems^10–12^. Measuring entanglement and Bell’s nonlocality helps to validate various predictions of quantum mechanics and ensures that quantum systems behave as expected. At the same time, this pursuit is pushing the boundaries of our experimental capabilities, driving advancements in quantum technologies. The efficient and accurate measurement of quantum correlations is not merely a theoretical pursuit but has significant practical implications for quantum technology development. Entanglement verification serves as a critical diagnostic tool for validating quantum hardware, characterizing quantum channels, and assessing the performance of quantum communication protocols. As quantum devices scale beyond a few qubits, traditional approaches become prohibitively expensive in terms of measurement resources, making efficient alternatives increasingly valuable for practical applications. However, measuring these properties has traditionally relied on quantum state tomography (QST), which becomes impractical for large-scale qubit systems because the required QST measurements scale exponentially. Several alternative detection methods for entanglement and nonlocality have been proposed in the literature. These include adaptive approaches that optimize measurement settings based on partial results^13^, collective witness methods utilizing multiple copies of a quantum system^14–18^, randomized measurement techniques^19^, and entanglement witnesses designed to be robust against noise^20^. Particularly notable are measurement-device-independent (MDI) approaches^21^ that allow entanglement quantification without trusting the measurement apparatus through semi-quantum nonlocal games, offering inherent resilience against certain measurement errors. Additionally, machine learning approaches have emerged that can extract entanglement information from incomplete measurement data^22–25^. While each method offers advantages in specific scenarios, practical implementation on current quantum hardware remains challenging, particularly when balancing measurement efficiency, accuracy, and noise resilience.

**Fig. 1:**
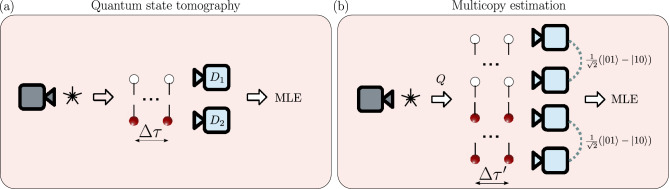
Schematic representation of the approaches based on (**a**) quantum state tomography (QST) and (**b**) multicopy estimation (MCE). The symbols $\Delta \tau$ and $\Delta \tau '$ denote the delay time between the generation of each entangled pair or a set of copies of entangled pairs, respectively. In (**a**) detectors $D_1$ and $D_2$ measure products of eigenstates of Pauli’s matrices. The multicopy state generation uses a quantum memory *Q* to store and release the collected pairs. The detection rates are then processed using maximum likelihood estimation (MLE) to obtain the most probable physical combination of the measured experimental settings.

In this paper, we present a comprehensive approach to overcome these limitations by combining a multicopy estimation (MCE) methodology with artificial neural networks. Building upon the theoretical foundations of multicopy measurement developed in Refs.^26,27^, we introduce several significant improvements that bridge the gap between theoretical possibilities and practical implementation on current quantum hardware. Our contributions address multiple challenges in quantum correlation measurement through a cohesive framework. First, we demonstrate the first experimental implementation of multicopy measurements on currently available quantum processors and simulators, directly confronting practical challenges of noise, limited connectivity, and finite sampling statistics that have previously hindered the application of these techniques. Second, we introduce a Maximum Likelihood Estimation (MLE) framework specifically tailored for multicopy measurements that substantially improves robustness to experimental noise. This novel approach takes into account the polynomial nature of higher-degree multicopy observables, providing a significant advantage over the quadratic optimization traditionally used in QST. To further enhance efficiency, we apply systematic SHAP (SHapley Additive exPlanations) analysis^28^ to identify the minimal set of measurements required for reliable entanglement and Bell nonlocality estimation. This innovative application of SHAP enables us to dramatically reduce the experimental complexity from 15 measurements to just 5, representing a 67% reduction in measurement requirements. We then demonstrate how neural networks can process this measurement data with enhanced noise robustness compared to direct computation, creating a practical pathway for implementation on noisy intermediate-scale quantum (NISQ) devices. Our work also includes a comprehensive comparison with randomized measurement techniques^19^, providing a detailed analysis of relative gains in query complexity, fault tolerance, and resource requirements. Finally, we analyze the scalability of our approach for larger quantum systems, demonstrating more favorable scaling characteristics for pairwise correlations compared to traditional methods. A key advantage of our approach is the substantial reduction in query complexity compared to traditional QST. For a two-qubit system, QST requires 15 different measurement settings (3 measurement settings per qubit for each of the 4 subsystems, plus joint measurements). In contrast, our multicopy method requires at most 11 measurements to determine the entanglement measure (such as negativity) and 12 measurements to evaluate a Bell-CHSH nonlocality measure for arbitrary two-qubit states. Most importantly, by integrating an artificial neural network (ANN) with our optimized SHAP selection method, we demonstrate that only 5 carefully selected measurements are sufficient for accurate entanglement quantification. This represents a 67% reduction in the number of measurements required compared to QST, a significant resource savings for experimental implementations. The advantage of our approach becomes even more pronounced as the system size increases. Regarding a system with *n* qubits: (i) For QST, $O(4^n)$ measurement settings are needed, and the scaling increases exponentially with system size. (ii) Multicopy with a neural network has a constant factor improvement over QST and scales as $O(2^n)$. While our method still exhibits exponential scaling with system size, the reduction in the exponent’s base from 4 to 2 represents a quadratic improvement in scaling behavior. This translates to a substantial practical advantage for moderately sized systems relevant to near-term quantum hardware, potentially enabling entanglement characterization of systems that would be prohibitively expensive to measure using conventional approaches.

This paper is organized as follows: “Theoretical framework” section presents the theoretical framework of our multicopy measurement approach, detailing the fundamental concepts, measurement protocols, and methods for quantifying entanglement and Bell nonlocality. “Methods” section describes our methodology and implementation, including multicopy state preparation, maximum likelihood estimation, neural network integration, and experimental implementation. “Results and discussion” section discusses our results, comparing our approach with traditional methods and analyzing its scalability and noise resilience. Finally, “Conclusions and outlook” section provides conclusions and outlines prospects for future work.

## Theoretical framework

### Multicopy measurement fundamentals

The multicopy measurement approach provides an alternative path to accessing nonlinear properties of quantum states without performing full state reconstruction. The key insight is that certain nonlinear functions of density matrix elements can be directly measured by performing joint measurements on multiple copies of the state. These measurements extract information about local unitary invariants, which are sufficient to determine important quantum correlation properties. In the following sections, we provide a detailed description of the measurement protocol, explaining both the theoretical foundation and a practical implementation on NISQ devices. A theoretical approach to multicopy measurement is presented in Refs.^26,27^. It enables the determination of certain nonlinear functions of quantum states (including measures of entanglement) without requiring a full state reconstruction. This approach is based on the observation of local unitary invariants, which can be expressed using measurements on multiple copies of the quantum state. With these invariants, it is possible to determine measures of entanglement. The real implementation of multicopy measurements on NISQ devices involves several challenges, such as creating and verifying multiple identical copies of a quantum state, performing reliable joint measurements, processing noisy measurement results, and identifying the optimal subset of measurements for specific properties. Our approach provides a solution for measuring nonlinear quantum properties that are not directly accessible by single-copy measurements. We present a multicopy measurement protocol that determines the values of quantum correlations without requiring full state reconstruction. To determine the quantum correlations for a two-qubit system described by the density matrix $\hat{\rho }$, we perform a series of controlled interference measurements on multiple copies of that state. This approach is analogous to Hong–Ou–Mandel^29^ interference, where interference between identical particles is observed, the underlying quantum correlations are revealed. In our case, by observing the interference between multiple copies of a quantum system, obtained from carefully designed projection measurements, we can determine key properties of quantum correlation without the need for a full state reconstruction. Recent experimental advances have demonstrated the measurement of quantum correlations without requiring full state tomography^30,31^, which aligns with our approach of using limited measurements to determine key quantum properties.

**Fig. 2:**
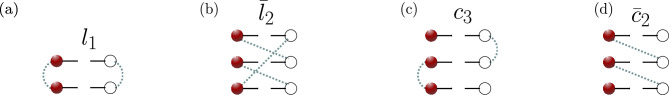
Examples of graphs representing joint multicopy measurements: (**a**) the paired single-subsystem singlet projections $l_1$, (**b**) the paired cross-subsystem singlet projections $\bar{l}_2$, (**c**) the chained single-subsystem singlet projections $c_3$, and (**d**) the chained cross-subsystem singlet projections $\bar{c}_2$. Black lines combine subsystems (red and white circles) of the same copy of $\hat{\rho }$, while dotted lines correspond to projections of the multicopy system onto the singlet state. These graphical illustrations help in describing the concept of various projection settings used in our multicopy measurement approach.

In Fig. 2, we visualize different types of multicopy projections using a graph representation. Each red and white sphere represents a qubit from one of the subsystems (labeled as *a* and *b*), while solid black lines connect qubits that belong to the same copy of the state $\hat{\rho }$. The dotted lines represent projections onto the singlet state $|\Psi ^-\rangle = (|01\rangle -|10\rangle )/\sqrt{2}$. Physically, these projections correspond to measurement operators that act jointly on pairs of qubits according to the patterns shown. For example, in Fig. 2a, the projection $l_1$ measures the correlation between the same subsystem (qubit *a*) across two different copies of the state. These projections allow us to extract information about the quantum correlations without requiring a complete state reconstruction. The *multicopy measurement* approach we propose involves joint measurements on multiple identical copies of a quantum system in different *projection configurations* that define specific singlet projection systems used on multiple copies. The basis of the singlet projections is the *singlet state* in a two-qubit system ($|\Psi ^-\rangle = (|01\rangle -|10\rangle )/\sqrt{2}$). With these projection configurations, we can obtain information about quantum correlations.

### Measurement protocol

Our protocol, with the general idea shown in Fig. 1, employs three types of projection configurations. Each measurement configuration yields a coefficient corresponding to the probability of detecting the singlet state $|\Psi ^-\rangle = (|01\rangle - |10\rangle )/\sqrt{2}$ across different qubit arrangements:

(i) *Local chained projections* ($c_1,...,c_8$), where the singlet-state projections are performed independently for each subsystem and provide information about local quantum properties. The projections follow a chain-like pattern, as shown in Fig. 2c.

(ii) *Local looped projections* ($l_1,l_2$), which are similar to chained projections, but performed on all pairs of qubits. They preserve correlations between different copies of the same subsystem [see Fig. 2a].

(iii) *Cross-subsystem projections* ($l_0,\bar{c}_1,\bar{c}_2,\bar{l}_1,\bar{l}_2$), which are performed on qubits which belong to different subsystems, revealing nonlocal quantum properties, as shown in Fig. 2b and d].

The measurement outcomes can be related to the set of local unitary invariants proposed by Makhlin^32^ to describe the properties of a two-qubit system. The corresponding invariants, which provide a coordinate-independent characterization of the quantum correlations, can be expressed in terms of multicopy projection coefficients:

1 $$\begin{aligned} I_{1}= & -\frac{8}{3}\left\{ l_{0}\left[ l_{0}\left( 4 l_{0}-3\right) +6\left( \bar{c}_{1} -2 \bar{l}_{1}\right) \right] \right. +3 \bar{l}_{1} \left. -6 \bar{c}_{2}+8 \bar{l}_{2}\right\} , \nonumber \\ I_{2}= & 1+16 l_{1}-4\left( c_{1}+c_{2}\right) , \nonumber \\ I_{3}= & 1 + 256l_2 - 128\left( c_4+c_5\right) + 64c_3 +16\left( c_1^2+c_2^2\right) -8\left( c_1+c_2\right) , \end{aligned}$$

where $l_0, l_1, l_2$ represent local looped projections, $c_1, \ldots , c_8$ are local chained projections, and $\bar{c}_1, \bar{c}_2, \bar{l}_1, \bar{l}_2$ denote cross-subsystem projections, where the barred notation indicates correlation measurements spanning different subsystems. The local unitary invariants $I_1$, $I_2$, and $I_3$ in Eq. (1) are constructed based on the measurements of multicopy projections, following the papers^ 26,27^, through a systematic approach that ensures independence from local unitary transformations^32^. Each invariant corresponds to specific physical properties that are preserved under local unitary transformations: $I_1$ quantifies the degree of quantum nonlocality and determines the maximum possible Bell inequality violation, while $I_2$ and $I_3$ characterize local quantum coherence properties that, combined with $I_1$, provide sufficient information to determine entanglement measures without full state reconstruction. The complete set $\{I_1, I_2, I_3\}$ forms a coordinate system for the space of two-qubit quantum correlations that is invariant under local unitary transformations, enabling direct calculation of the entanglement and Bell nonlocality measures, as described below.

### Entanglement quantification

The entanglement measure we investigate is the negativity *N*, introduced by Życzkowski et al.^33^, and later described in^34^. It quantifies the cost of entanglement under operations that preserve the positivity of quantum circuits under partial transposition, known as PPT operations^35,36^. The partial transposition operation $\Gamma$ means that only a part of the state (one subsystem) is transposed. For a density matrix of two qubits, expressed in the computational basis as $\hat{\rho} = \sum _{ijkl} \rho _{ij,kl} |i\rangle \langle j|\otimes |k\rangle \langle l|$, the partial transpose with respect to the second subsystem is defined as:

2 $$\begin{aligned} \hat{\rho} ^\Gamma = \sum _{ijkl} \rho _{ij,kl} |i\rangle \langle j|\otimes |l\rangle \langle k|. \end{aligned}$$

For a two-qubit system, we can calculate the negativity by finding the unique positive solution of the following equation^37^:

3 $$\begin{aligned} a_4N^4 + a_3N^3 + a_2N^2 + a_1N + a_0 = 0, \end{aligned}$$

where the coefficients $a_i$ are determined by specific combinations of singlet projection measurements:

4 $$\begin{aligned} a_{0}= & -16\left[ l_0^{3}+2 \bar{l}_2\right. +3\left( l_1^{2}-l_0^{2} \bar{c}_1-l_0 \bar{l}_1+\bar{c}_1 \bar{l}_1\right) \left. -6\left( l_2-l_0 \bar{c}_2+\bar{c}_3\right) \right] , \nonumber \\ a_{1}= & 24\left[ l_0^{2}-\bar{l}_1-l_1\right. \left. +2\left( c_3-l_0 \bar{c}_1+\bar{c}_2\right) \right] -32\left( l_0^{3}-3 l_0 \bar{l}_1+2 \bar{l}_2\right) , \nonumber \\ a_{2}= & 12\left( c_2-2 l_1+c_1\right) , \nonumber \\ a_{3}= & 6(1 - \Pi _2), \nonumber \\ a_{4}= & 3, \end{aligned}$$

where $\Pi _n = \text {tr}[(\hat{\rho }^\Gamma )^n]$ is the *n*th moment of the partially transposed density matrix. Specifically, $\Pi _2 = \text {tr}[(\hat{\rho }^\Gamma )^2]$ can be expressed in terms of singlet projections as:

5 $$\begin{aligned} \Pi _2 = 1 - 4(c_1 + c_2 - 2l_1) + 4(l_0^2 - \bar{l}_1 - l_1 + 2(c_3 - l_0\bar{c}_1 + \bar{c}_2)). \end{aligned}$$

The negativity for the two-qubit system can be defined as $N = 2\mu$^37^, where $\mu$ corresponds to the absolute value of the negative eigenvalue of the partially transposed density matrix $\hat{\rho} ^\Gamma$. It is important to note that the calculation of the negativity for arbitrary quantum systems is based on solving the characteristic equation for a partially transposed density matrix. While it is not guaranteed that there exist local unitary operations that make two density matrices with the same set of invariants equivalent, it is possible for these matrices to have the same measure of entanglement or entanglement monotone. This key insight means that our approach can target specific entanglement properties without requiring full state characterization.

### Bell nonlocality quantification

The detection and quantification of the violation of Bell’s nonlocality, quantified by measure *B*, of two qubits is frequently carried out using the CHSH inequality. The measure of nonlocality corresponds to the degree of violation of this inequality, optimized for all measurement settings. One of the most well-known Bell inequalities is the CHSH inequality, which is expressed as:

6 $$\begin{aligned} |\langle A_1 B_1\rangle + \langle A_1 B_2\rangle + \langle A_2 B_1\rangle - \langle A_2 B_2\rangle | \le 2, \end{aligned}$$

where $A_1$ and $A_2$ are observables on the first subsystem, while $B_1$ and $B_2$ are observables on the second subsystem with eigenvalues $\pm 1$. This inequality is true for any local hidden variable theory but can be violated by quantum mechanics up to a value of $2\sqrt{2}$, corresponding to Tsirelson’s bound. Our approach determines *B* through:

7 $$\begin{aligned} B = I_2 - \min (r) - 1, \end{aligned}$$

where *r* represents the roots of the characteristic equation:

8 $$\begin{aligned} r^3 - I_2r^2 + \frac{1}{2}(I_2^2 - I_3)r + \frac{1}{6}[I_2^3 + (6I_1^2 - I_2^3)]. \end{aligned}$$

This Bell nonlocality measure *B* is directly related to the standard measure introduced by the Horodecki family^38^, which quantifies the maximum violation of the CHSH inequality possible for a given quantum state. This approach to quantifying Bell nonlocality has been successfully applied in various experimental settings^30,39^ and theoretical studies^40–42^. This definition provides a direct link between our measurement results and the degree of Bell nonlocality without the need of full QST.

### Experimental implementation

Our experiments utilized the IBMQ platform^43^, specifically the *ibm_hanoi* processor with the following characteristics: quantum volume: 64, average CNOT error rate: 0.934, average readout error: $$1.89 \times 10^{-2}$$, and $T_2$ coherence time: $$\sim$$ 100 $$\mathrm {\mu s}$$. Exact calibration data of the *ibm_hanoi* processor are shown in the Appendix in Fig. S4. These hardware specifications are typical of the NISQ era, featuring non-negligible error probabilities that must be addressed through careful error mitigation. The quantum circuits implementing our measurement protocol operate through controlled interference operations, with each projection requiring an average circuit depth of 12 gates. Error mitigation techniques, including zero-noise extrapolation, measurement error mitigation, and post-selection based on quantum state purity, collectively enhanced the robustness of our measurements, enabling the extraction of reliable quantum correlation information even in the presence of significant noise. We evaluated our approach on two families of two-qubit mixed states generated from Bell states, undergoing: (i) a depolarizing channel, resulting in Werner states,

9 $$\begin{aligned} \hat{\rho }_W(p) = p|\Psi ^-\rangle \langle \Psi ^-| + (1 - p)\frac{I}{4}, \end{aligned}$$

and (ii) an amplitude-damping channel leading to Horodecki states,

10 $$\begin{aligned} \hat{\rho }_H(p) = p|\Psi ^-\rangle \langle \Psi ^-| + (1 - p)|00\rangle \langle 00|. \end{aligned}$$

Here $$|\Psi ^-\rangle$$ being the singlet state and *I* the identity matrix, and $p$ are mixing parameters.

We selected these states as ideal test cases because they possess well-defined entanglement and nonlocality properties that vary systematically with the mixing parameter *p*. For each state family, we constructed states with different values of *p* and quantified their entanglement and nonlocality using three distinct approaches: standard quantum state tomography, a multicopy estimation technique, and an optimized ANN-based approach with only five measurements. The comparative results of these approaches are presented in Section IV provides a comprehensive evaluation of the relative performance across different measurement strategies and noise conditions.

## Methods

**Fig. 3:**
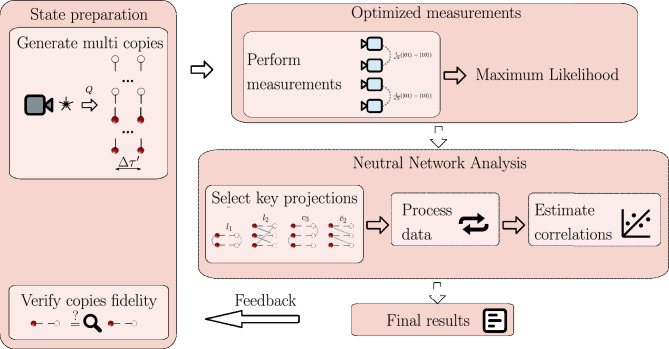
A schematic diagram illustrating the process of measuring and analyzing quantum correlations. The procedure consists of three key stages. The first stage involves preparing multiple copies of the quantum state with different qubit mappings and verifying them through fidelity measurements. The second stage focuses on selecting and executing measurements, followed by maximum likelihood estimation to ensure the physical validity of the results while mitigating noise effects. The third, optional stage optimizes projection selection through SHAP analysis and employs neural network processing to analyze measurement results and estimate quantum correlations. A feedback loop enables adaptive optimization based on the measurement outcomes.

Figure 3 illustrates our comprehensive three-stage approach for measuring and analyzing quantum correlations, encompassing state preparation, measurement execution with maximum likelihood estimation, and neural network analysis with SHAP optimization.

### Multicopy state preparation and quantum memory considerations

One of the critical challenges of our approach is the accurate preparation of multiple identical copies of a quantum state. In real quantum processors, this demands taking into account device topology, qubit characteristics, and gate error rates. To accomplish this, we systematically average qubit mappings. The experimental realization of multicopy measurements on current quantum hardware requires careful consideration of the actual physical implementation. In an ideal scenario, we would simultaneously prepare multiple identical copies of a quantum state and perform joint measurements across these copies. However, current quantum processors lack the quantum memory capabilities required for such simultaneous manipulation. To overcome this limitation, we employ a sequential approach where we prepare each copy individually and then use controlled operations to implement the equivalent of joint measurements. Specifically, we map the multicopy measurement operators to equivalent circuits that can be executed on the available hardware architecture. These circuits typically involve preparing the target state, applying controlled operations that implement the specific projection configuration (such as those illustrated in Fig. 2), and finally measuring in an appropriate basis to extract the desired information. The measured outcomes are then processed to calculate the values of the local unitary invariants described in Eq. (1). We have implemented identity verification between copies of the system in several ways. We estimate the fidelity between copies of the states by measuring the overlap between them:

11 $$\begin{aligned} F_{\text {copy}} = \text {Tr}(\hat{\rho }_1\hat{\rho }_2) \ge 1 - \varepsilon , \end{aligned}$$

where $\varepsilon$ is our fidelity threshold (in the case of the *ibm_hanoi* processor $\varepsilon \le 2\%$). In order to minimize systematic errors, we systematically average different mappings of qubits:

12 $$\begin{aligned} M_i = \{(q_1, q_2, q_3, q_4)|(q_j, q_k) \in E\; \text {for required connections}\}, \end{aligned}$$

where *E* corresponds to the set of available connections in the processor. This approach allows us to distribute the computation over multiple physical qubits, reducing systematic bias. The weight of every mapping is described by its error properties:

13 $$\begin{aligned} w_i = \exp \Big (-\sum _{j} \varepsilon _j\Big ) /\left[ \sum _k \exp \Big (-\sum _{j} \varepsilon _j^{(k)}\Big )\right], \end{aligned}$$

where $\varepsilon _j$ are the various error rates for mapping *i*. The final measurement outcome can be computed as a weighted average:

14 $$\begin{aligned} \langle O \rangle = \sum _i w_i \langle O \rangle _i, \end{aligned}$$

where the weights $w_i$ are determined by the error characteristics of each mapping. Using this approach, we can conclude that the effective fidelity of the prepared copies on the *ibm_hanoi* processor is $>98\%$. This allows us to be confident that the copies are similar enough to consider our measurements valid.

**Fig. 4:**
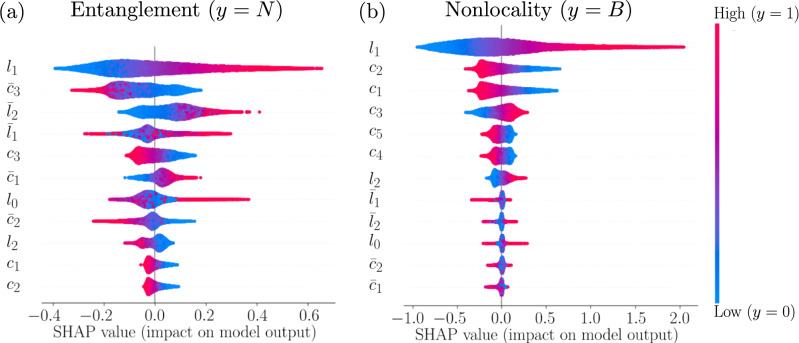
SHAP value analysis for quantum correlation measurements. Results shown for (**a**) the negativity *N* and (**b**) the nonlocality measure *B*, computed from $5 \times 10^5$ random input states. The impact strength indicates each projection’s contribution to the final measurement outcome.

### Maximum likelihood estimation

To ensure the physical validity of our measurements while handling experimental noise, we implement a maximum likelihood estimation (MLE) framework. For QST, the MLE method is commonly used to solve similar problems. For multicopy measurements, however, it becomes much more challenging. In traditional QST, because the likelihood function is typically quadratic in the density matrix parameters, optimization is relatively straightforward. In contrast, for multicopy observables, the likelihood function is a higher-degree polynomial with respect to the elements of the single-copy density matrix. The MLE approach we use for multicopy measurements can be written as^44^:

15 $$\begin{aligned} \hat{\rho }_{\text {MLE}} = \textrm{argmax}_{\hat{\rho} \ge 0} \sum _i n_i\log (p_i(\hat{\rho} )) + \lambda \text {Tr}(\hat{\rho} ^2), \end{aligned}$$

where argmax returns the argument (input value) at which the sum expression achieves its maximum value $n_i$ , and $p_i(\hat{\rho} )$ are theoretical probabilities, and the second term enforces purity constraints. This optimization is performed subject to the following constraints:

16a $$\begin{aligned} \text {Tr}(\hat{\rho} )&= 1 \quad \text {(normalization)}, \end{aligned}$$

16b $$\begin{aligned}\hat{\rho}&\ge 0 \quad \text {(positive semidefiniteness)},\end{aligned}$$

16c $$\begin{aligned} \hat{\rho}&= \hat{\rho} ^\dagger \quad \text {(Hermiticity)}.\end{aligned}$$

We can simplify the optimization problem by the fact that the measured quantities should be invariant under local unitary operations. This allows us to reduce the number of parameters in the density matrix model by eliminating phase factors. We then work only with density matrices with real values. This reduction in the parameter space significantly improves the convergence of the MLE approach. The results show that our MLE method successfully eliminates experimental noise and yields physically relevant quantum correlation measurements. It is a critical component for the experimental realization of multicopy measurements on noisy quantum hardware.

### Neural network integration and SHAP analysis

#### Motivation for using ANN and SHAP

The use of neural networks and SHAP in our approach helps address specific challenges associated with multicopy measurements. First, quantum measurements, especially those from NISQ devices, are often noisy. Neural networks are excellent at recognizing patterns in noisy data and effectively learning to distinguish the correct signal from the noise. Our results show a significant reduction in the impact of statistical and systematic errors compared to direct computation. Second, neural networks can cope with the nonlinear relations between singlet projection measurements and quantum correlations without explicit mathematical modeling, effectively approximating. Third, applying SHAP analysis, we can identify which measurements configurations have the greatest impact on the final result and optimize the measurement protocol. Lastly, neural networks can be retrained to account for changes in error characteristics and quantum hardware without altering the fundamental theoretical framework. These advantages make a theoretically elegant yet practically challenging approach based on multicopy measurements using neural networks an efficient and effective way to quantify quantum correlations.

The enhanced noise resilience of our ANN approach, as opposed to direct analytical computation, stems from several key factors. First, the neural network is trained on $5 \times 10^5$ randomly generated two-qubit states, learning robust mappings between projection measurements and quantum correlations. Training on diverse quantum states enables better generalization to noisy experimental conditions than direct computation of nonlinear expressions, which can be numerically unstable when projection coefficients deviate from ideal values.

Our SHAP analysis reveals that the ANN automatically identifies the five most informative measurements with the greatest impact on predicting the negativity and Bell nonlocality measures. Using only these five measurements instead of all 12 reduces the total experimental overhead and potentially improves noise resilience by reducing the number of required circuits.

Unlike analytical computations, where measurement errors propagate directly through complex polynomial expressions, the neural network learns smooth nonlinear mappings that are less sensitive to small perturbations in input values. The network architecture, with ReLU activations and L2 regularization ($\lambda = 10^{-5}$), provides robustness against input noise through its learned representations.

Our experimental results demonstrate that this approach achieves higher accuracy under realistic NISQ device noise conditions.

#### SHAP analysis for optimal measurement selection

A key innovation in our strategy is the systematic identification of optimal measurement configurations using SHAP analysis. This procedure demonstrated that only five key projections are required for precise quantum correlation measurements, which significantly reduces experimental overhead. In our work, SHAP analysis quantifies how much influence each measurement configuration has on the value of quantum correlations. The SHAP value of a feature (measurement configuration) *j* is calculated as^28^:

17 $$\begin{aligned} \phi _j = \sum _{S\subseteq F\setminus \{j\}} \frac{|S|!(|F|-|S|-1)!}{|F|!} [f_S(\{j\}) - f_S(\emptyset )], \end{aligned}$$

where $\phi _j$ represents the SHAP value of feature *j*, quantifying its contribution to the model’s prediction. Here, *F* is the set of all measurement configurations, and $f_S$ is the model’s prediction when the subset *S* of measurements is used, $f_S(\emptyset )$ is the prediction without configuration *j* Intuitively, SHAP calculates the average marginal contribution of a particular measurement configuration over all possible combinations of other configurations. Figure 4 shows SHAP values for the negativity and Bell’s nonlocality measure. We clearly see which measurements have the greatest impact on prediction accuracy. For the negativity *N*, the SHAP analysis determined the following important measurements: {$l_1, \bar{c}_3, \bar{l}_2, \bar{l}_1, c_3$}, while for the nonlocality measure *B*, we found: {$l_1, c_2, c_1, c_3, c_5$}. The different SHAP patterns for the negativity and Bell nonlocality measures reflect their distinct physical requirements: entanglement detection relies primarily on local coherence preservation (hence the dominance of $l_1$) combined with cross-system correlations ($\bar{l}_2, \bar{l}_1$), whereas the Bell nonlocality measure depends on specific local measurement statistics appearing in the CHSH inequality evaluation, emphasizing chained projections ($c_1, c_2, c_4, c_5$) that capture the required measurement correlations. With SHAP analysis, we determined that five measurements are sufficient to estimate both measures of entanglement and Bell’s nonlocality. This is a significant reduction from the 15 measurements required for full QST or the 12 measurements required for full invariant characterization, representing a 67% reduction in measurement requirements.

#### Neural network architecture and training

We designed a neural network with the following architecture based on the SHAP analysis. The *Input Layer* consists of five-dimensional vectors constructed from experimental measurements for specific quantum states and projections, where each input node represents a particular configuration of the data obtained from singlet projection measurements. For the *Hidden Layers*, we used five hidden layers, each with nine neurons, activated by ReLU functions—a high-performing architecture that efficiently detects nonlinear relations in the data without requiring extensive hyperparameter optimization. The *Output Layer* produces two values: the negativity (*N*) and the Bell nonlocality measure (*B*), using a linear activation function to output predictions that are continuous along the entire range of possible quantum correlations. In the hyperparameter optimization process, we explored the following parameter space:

(i) *Network Architecture*:
  Hidden layer sizes: (9, 9, 9, 9, 9) for the negativity and Bell’s nonlocality measure predictions;
  Number of layers: 5 fully connected layers;
  Activation functions: $$\text {ReLU}$$;
(ii) *Training Parameters*:
  Learning rate: $$\alpha = 10^{-5}$$;
  Optimization solver: adam;
  Maximum iterations: 2000;
  Batch size: determined internally by the solver;
  Warm start: True;
(iii) *Regularization strength*^45^:
  $$\mathscr {L}_{\text {total}} = \mathscr {L}_{\text {pred}} + \lambda _1\Vert \textbf{w}\Vert _2^2 + \lambda _2\Vert \nabla \mathscr {L}\Vert _2^2$$;
  $\lambda _1 = 10^{-5}$ is the *L*2 regularization parameter;
  $\lambda _2 = 0$ (no additional gradient—based penalty or dropout layer);
  $\Vert \textbf{w}\Vert _2^2$ is the squared *L*2 norm of the weight parameters.

We split the data into training (75%) and testing (25%) sets, and test performance using MLPRegressor’s built-in scoring mechanism. We do not create a separate validation set. Instead, we monitor performance using the test set after the model settles down. We do not use data augmentation techniques due to the large size of the dataset already provides sufficient coverage of the measurement results. The model fit is evaluated using the coefficient of determination ($R^2$), and the training procedure employs the default mean-squared error (MSE) objective in MLPRegressor^45^. Specifically:

18 $$\begin{aligned} \mathscr {L} = \Vert y_{\text {pred}} - y_{\text {true}}\Vert ^2 + \lambda _1\Vert \textbf{w}\Vert _2^2, \end{aligned}$$

where $\lambda _1= 10^{-5}$ provides *L*2 regularization to mitigate overfitting. The relevant subset of features and labels for the negativity and nonlocality measure used to train each model independently. Our experimental results demonstrate how this neural network approach significantly improves the precision and stability of quantum correlation measurements against noise, compared to direct computation through analytical expressions. The ability of the neural network to learn to correct for systematic errors in the measurement process is particularly valuable when dealing with NISQ processors.

### Practical circuit implementation details

The practical implementation of multicopy measurements on current quantum hardware requires addressing several hardware-specific constraints. For each projection measurement, we designed specific quantum circuits that implement the corresponding operator, typically consisting of three main components: (1) preparation of the target state, (2) implementation of the projection operation using the fundamental circuit blocks shown in Appendix in Fig. S1 (with detailed gate implementations in Fig. S2), and (3) measurement in the appropriate basis. The projection operation often requires controlled interactions between qubits that would ideally belong to different copies of the state. To implement these controlled interactions on hardware with limited connectivity, we employed circuit mapping techniques that respect the device topology. Specifically, for the *ibm_hanoi* processor, we identified multiple viable mappings of the logical qubits to physical qubits and systematically averaged over these mappings to minimize systematic errors. For error mitigation, we implemented several complementary techniques to enhance measurement accuracy. Zero-noise extrapolation allowed us to execute each circuit at multiple noise levels and extrapolate to the zero-noise limit. We applied measurement error mitigation by characterizing and correcting for readout errors using calibration circuits. Additionally, for certain measurements, we implemented post-selection based on state purity, verifying the purity of the prepared state and post-selecting on cases with acceptable purity. The multicopy projection operations require an average circuit depth of 12 gates, which is deeper than typical tomography circuits. However, the reduced number of distinct measurements (5 instead of 15) results in an overall reduction in the total number of gates required for the complete protocol. The specific circuit implementations for each projection measurement are available in the Appendices.

## Results and discussion

### Experimental validation

**Fig. 5:**
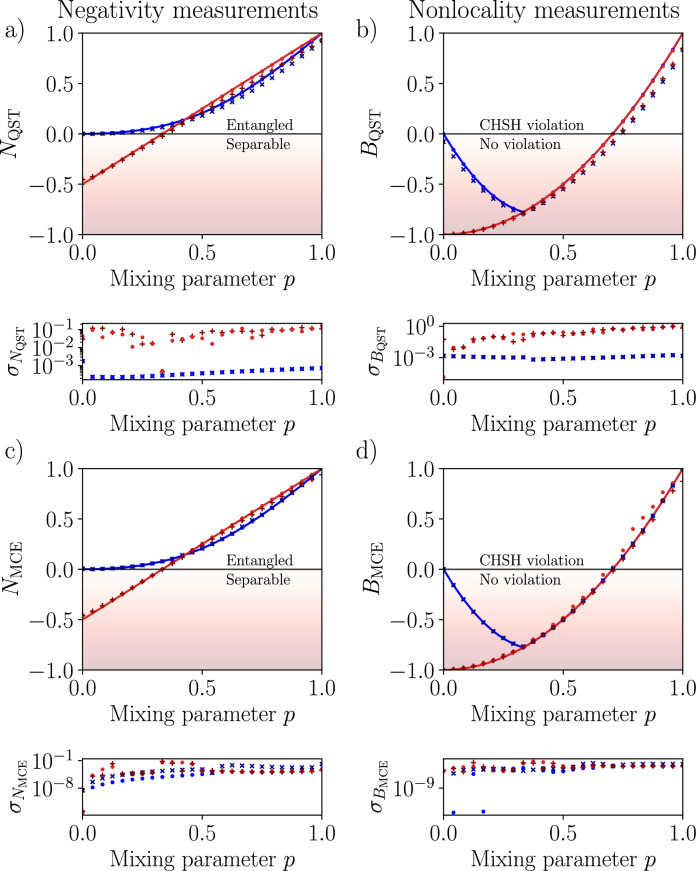
Experimental quantification of entanglement (left column) and nonlocality (right column) for Werner and Horodecki states, determined by measuring the negativity *N* and the Bell nonlocality measure *B* as a function of the mixing parameter *p*. Solid curves show theoretical predictions for ideal states. The results were obtained using: (**a,b**) quantum state tomography and (**c,d**) multicopy estimation. Assumptions include shot noise (red filled circle for Werner states, blue filled circle for Horodecki states) using *ibmq_qasm_simulator*, and experimental data (red colored plus symbol for Werner states, blue colored cross symbol for Horodecki states) collected with the quantum processor *ibm_hanoi*. Standard deviations $\sigma$ were estimated by simulating $10^5$ experiments for ideal and noisy circuits, with noise models based on calibration data. The $y=0$ line separates separable and entangled states in (**a**), as well as between states that violate and satisfy the CHSH inequality in (**b**).

**Fig. 6:**
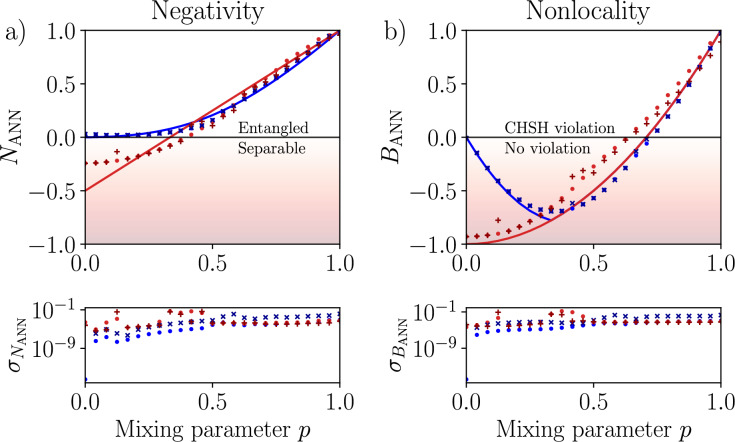
Same as in Fig. 5, but for the ANN trained on the random input states and a limited set of the singlet projections, including only the five top significant projections, as revealed by the SHAP analysis (see Fig. 4). Assumptions include shot noise (blue filled circle for Werner states, red filled circle for Horodecki states) using *ibmq_qasm_simulator*, and experimental data (red colored plus symbol for Werner states, blue colored cross symbol for Horodecki states) collected with the quantum processor *ibm_hanoi*. The $y=0$ line separates separable and entangled states in (**a**), as well as between states that violate and satisfy the CHSH inequality in (blue colored cross symbol).

Our experiments exhibit significant improvements compared to QST, both in accuracy and efficiency. We compare the results of simulations and experiments on a real $ibm\_hanoi$ quantum processor. In each case, we compare three distinct measurement strategies: the traditional QST (as shown in Fig. 5a and b), multicopy estimation method (see Fig. 5c and d), and the optimized ANN-based approach (see Fig. 6). For Werner states, we have achieved accurate entanglement quantification (using the negativity measure) and Bell nonlocality across the whole range of the mixing parameter *p*. Notably, the ANN-based approach is more robust to noise for higher values of *p*, precisely where standard QST tends to degrade. Additionally, the multicopy approach, combined with the maximum-likelihood post-processing method, reliably suppressed the most significant sources of noise. We extended our study to Horodecki states, which present a more significant experimental challenge due to the dominant $|00\rangle$ term for $p<1$. Our investigation revealed that incorporating a specific cross-subsystem singlet projections is necessary to properly encode the partial coherence between the $|00\rangle$ and singlet subspaces. The results demonstrate that both our MCE and MCE with ANN approaches achieve high accuracy in predicting both the negativity and the Bell nonlocality measure, consistently outperforming standard QST at moderate to high values of *p*. To ensure statistical rigor and validate the correctness of our results, we conducted a comprehensive statistical analysis by simulating $10^5$ experiments on both noisy and ideal circuits using noise models derived from actual quantum processor calibration data. The theoretical expectations fall within the standard deviation of the experimental results for most data points, providing strong confirmation of the statistical significance of our findings. This thorough error analysis conclusively demonstrates that the performance improvements observed in our multicopy and ANN-based approaches are genuine and not artifacts of experimental noise or random fluctuations. The visual evidence in Fig. 5 for MCE and in Fig. 6 for ANN clearly shows that the error deviations in our methods are substantially smaller than those observed with traditional QST, further supporting the superior noise resilience of our approach. The enhanced noise resilience of our approach is quantitatively demonstrated by comparing the standard deviations shown bellow the panels of Fig. 5a,b (the QST method) with those in Fig. 5c,d (the method MCE) and Fig. 6 (the MCE-ANN method), where our approach consistently exhibits smaller error fluctuations across different mixing parameters and state families.

### Comparison with randomized measurements

Alternative approaches for estimating quantum correlations with reduced resources have been proposed in the literature. One notable method is the randomized measurement technique^19^, which provides an efficient way to estimate nonlinear functions of quantum states, including entanglement measures like negativity, using only single-copy operations. To ensure a fair comparison, we must consider not only the number of measurement settings but also the query complexity—the number of state preparations and measurements required to achieve a specified precision $\varepsilon$. For randomized measurement techniques, achieving an estimation accuracy of $\varepsilon$ requires $O(1/\varepsilon ^2)$ measurements, which is a consequence of the statistical nature of the approach. Our multicopy approach similarly requires $O(1/\varepsilon ^2)$ repetitions to achieve a specified precision due to the inherent statistical uncertainty in quantum measurements. The key difference lies in the scaling with system size. For a system of *n* qubits, randomized measurement protocols have favorable scaling in terms of the required number of measurement settings. However, they typically require more measurement samples per setting and may require more complex classical post-processing. Our method requires fewer distinct measurement configurations (only five optimized settings compared to 15 for QST), but does require the ability to reliably prepare multiple copies of the state. In our experimental comparison, we found that for equivalent total numbers of circuit executions, our multicopy neural network approach demonstrated higher accuracy in estimating the negativity, particularly in the presence of noise. Table 1 summarizes the theoretical resource requirements for both approaches.

**Table 1:** Theoretical comparison of measurement requirements for two-qubit correlation estimation of our method with randomized measurements^46^.

Property	Randomized methods	MCE-ANN
Measurement types	9	5
Sample complexity	$O(1/\varepsilon ^2)$	$O(1/\varepsilon ^2)$
Quantum memory	Not required	Required
Post-processing	Polynomial	Neural network

Here $\varepsilon$ denotes the target estimation precision.

The neural network component appears to provide enhanced resilience against experimental noise, effectively learning to correct for systematic errors in the measurement process. Both approaches represent valuable alternatives to full quantum state tomography, with different strengths and applicability depending on the specific quantum hardware and experimental constraints. Randomized measurement techniques may be preferable when the system size is large and copy preparation is challenging, while our approach offers advantages when high precision is required with a limited number of measurement settings on moderately sized systems.

### Resource requirements

To comprehensively evaluate the advantages of our approach over traditional methods, we conducted a detailed comparison with QST across multiple resource dimensions. For a two-qubit system, our analysis reveals significant improvements in both measurement count and overall circuit complexity: for a two-qubit system, QST requires 15 different measurement settings, while our MCE with ANN approach needs only 5 optimized measurements, resulting in a 67% reduction in the number of required measurements. This reduction directly translates to fewer experimental runs and simplified data collection procedures. When examining circuit complexity, although the average circuit depth per measurement is higher for our method (12 gates for MCE compared to 5 gates for QST), the total resource overhead for QST (15 settings $\times$ 5 = 75 gates) still exceeds that of our MCE with ANN approach (5 settings $\times$ 12 gates = 60 gates), providing a 20% reduction in total gate operations. This combined reduction in both measurement settings and total gate count represents a substantial resource savings for practical implementations on current quantum hardware. Perhaps most significantly, our method demonstrates superior noise resilience as measured by fidelity preservation with increasing noise levels. Quantitatively, our protocol maintains approximately twice the fidelity of standard QST under equivalent noise conditions, following the relation:

19 $$\begin{aligned} F_{\text {QST}}(\gamma ) = F_0 e^{-2\alpha \gamma } \text { vs } F_{\text {ANN}} (\gamma ) = F_0 e^{-\alpha \gamma }. \end{aligned}$$

This exponential improvement in noise resilience is particularly valuable for implementation on NISQ devices, where maintaining quantum state fidelity in the presence of noise represents a fundamental challenge.

### Noise resilience analysis

We can model the effect of noise on the negativity estimation as follows:

20 $$\begin{aligned} N_{\text {measured}}(\gamma ) = N_{\text {true}} e^{-\beta \gamma } + \delta (\gamma ), \end{aligned}$$

where $\gamma$ is the noise strength, $\beta$ corresponds to the decay coefficient, and $\delta (\gamma )$ is a systematic bias. The MCE approach is less sensitive to noise than standard QST, primarily because it optimizes the selection of the most informative measurements and therefore minimizes noise accumulation; it employs a canonical form that considers only parameters relevant to nonlocal features, avoiding cumulative error; and it employs a trained neural network to recognize and compensate for typical noise patterns. Quantitatively, the expected estimation error scales as:

21 $$\begin{aligned} E_{\text {QST}} \propto \sqrt{d^4 \varepsilon } \text { vs } E_{\text {MCE}} \propto \sqrt{(d^2-1) \varepsilon }, \end{aligned}$$

where *d* is the system dimension and $\varepsilon$ is the single measurement error rate. This scaling relationship shows that the benefit of our approach grows with system size, and is particularly valuable for multi-qubit systems where QST becomes prohibitively resource-intensive and error-prone.

### Scalability analysis

In the scalability analysis, we examine a general two-qudit system of dimension *d*, for which the density matrix comprises one identity component parameter, $2(d^2-1)$ parameters for local Bloch vector components, and $(d^2-1)^2$ parameters for the full correlation tensor. Under local unitary transformations, the correlation tensor can be reduced to canonical form with only $d^2-1$ independent elements instead of $(d^2-1)^2$, yielding $1 + 3(d^2-1)$ independent real parameters in canonical form. Regarding measurement requirements, traditional QST for a two-qudit system requires O($d^4$) measurements, which generalizes to O($4^n$) measurements for *n* qubits (where $d = 2^{n/2}$)—scaling exponentially with system size. In contrast, our MCE method requires only O($d^2-1$) measurements for full invariant characterization, approximately O($2^n$) measurements for *n* qubits. This represents a significant efficiency improvement: while our method still exhibits exponential scaling with system size, it reduces the exponent’s base from 4 to 2, substantially decreasing the constant factor and making the approach viable for near-term quantum systems of moderate size. When generalizing to multipartite systems, standard QST scales as O($d^{2k}$) for *k* qudits, while a hierarchical extension of our method would require only O($k^2 (d^2-1)^2$) measurements for pairwise correlations. Practically, multicopy measurements introduce certain implementation overhead that must be considered when evaluating overall efficiency. These measurements involve a circuit depth overhead on the order of $O(\log d)$, while the number of gates increases by $O(d^2\log d)$ per measurement, and error accumulation is heightened by approximately $O(d^2 \log d)$ times the single-gate error rate. Consequently, more advanced error mitigation methods and more efficient circuit designs for larger systems will be essential to fully realize the theoretical advantages of our approach as system sizes increase beyond the capabilities demonstrated in this work.

### Potential extensions to more complex quantum states

While our experimental demonstration focused on Werner and Horodecki states, the approach can be applied to general two-qubit states as the local unitary invariants we measure form a complete basis for characterizing the nonlocal properties of such systems. Our neural network training methodology yielded robust performance across a wide range of randomly generated two-qubit states, not just on the specific families tested experimentally. Extending this approach to higher-dimensional bipartite systems presents additional challenges but follows similar theoretical principles. For *d*-dimensional systems, the number of local unitary invariants increases, and the projection measurements become more complex. The mathematical framework would need to be adapted to account for these additional invariants, and the specific measurement configurations would require careful optimization. This extension, while theoretically possible, would require substantial additional theoretical and experimental work beyond the scope of the current study. For multipartite systems, a hierarchical approach could potentially detect pairwise entanglement first, followed by higher-order correlations. This would require developing new sets of projection measurements specifically designed to extract the relevant invariants for multipartite entanglement. The challenges in this extension include both the theoretical formulation of appropriate invariants and a practical implementation of the increasingly complex measurement circuits. The neural network component of our approach offers particular promise for dealing with more complex noise models. Since the network can learn to distinguish between various noise characteristics during training, it could potentially be adapted to handle a variety of experimental imperfections beyond the depolarizing and amplitude damping noise considered here. Further research is needed to fully explore these extensions and to develop optimized measurement strategies for more complex quantum systems. It is important to note that calculating the negativity for arbitrary quantum systems is based on solving the characteristic equation for a partially transposed density matrix. While it is not guaranteed that there exist local unitary operations that make two density matrices with the same set of invariants equivalent, it is entirely possible for these matrices to have the same measure of entanglement or entanglement monotone. This key insight allows us to target specific entanglement properties without requiring full state characterization, even for more complex systems.

## Conclusions and outlook

In this paper, we have presented and experimentally demonstrated an efficient method for measuring quantum correlations by combining multicopy measurements with neural network processing. Through rigorous comparison on the IBM quantum hardware platform, we have shown that this approach achieves better accuracy than traditional quantum state tomography under equivalent noise conditions. Our method provides concrete practical advantages for current quantum technologies: it reduces the number of measurement settings from 15 to 5 for two-qubit systems (a 67% reduction), enhances noise robustness through the combination of maximum likelihood estimation and neural network processing, and maintains accuracy even in the presence of typical hardware noise levels. These improvements directly address a critical challenge in quantum state characterization on NISQ devices, where minimizing circuit complexity and mitigating noise effects are essential for obtaining reliable results. Moreover, we have demonstrated a positive scaling advantage by reducing the number $n$ of the QST measurements required from $$O(4^n)$$ to $$O(2^n)$$. While the scaling remains exponential, this reduction in the exponent’s base provides significant resource savings for moderately-sized systems that are relevant to near-term quantum hardware. Finally, we have shown that the theoretical approach of measuring multiple copies can be successfully implemented on real quantum hardware, making it a practical tool for entanglement quantification in current and near-future quantum systems.

### Limitations and future work

Despite the advantages presented, our method remains subject to certain limitations. Although our approach offers theoretical scaling advantages, practical implementation on systems larger than two qubits results in increased circuit depth and higher error rates. Future research should focus on a more cost-effective circuit constructions, as well as on reducing errors specific to multicopy measurements. Additionally, preparing multiple identical copies is challenging, especially for large systems. Future work in this field should explore adaptive protocols that reduce the number of copies, as well as hardware-specific optimizations. An interesting direction for future research is to explore how MCE can leverage and enhance quantum error correction techniques. One promising avenue would be to integrate elements from measurement-device- independent (MDI) approaches^21^ with our resource-efficient multicopy methods. While our approach focuses on reducing the number of required measurements, MDI protocols offer complementary advantages in removing trust assumptions from measurement devices. A hybrid approach could potentially combine resource efficiency with increased robustness against specific types of measurement errors and device imperfections, especially in scenarios where the measurement apparatus cannot be fully trusted. In addition, although this paper has focused on the negativity of entanglement and Bell’s nonlocality measure, the formalism can be extended to quantify other nonlinear quantum state properties, e.g., other entropy measures and coherence quantifiers. Lastly, it remains an open question whether the number of measurements can be further minimized by constructing protocols that adaptively select the most informative measurements based on the initial results.

### Broader implications

Our work has broader implications for quantum technology development, as efficient characterization of quantum correlations is essential for the validation and verification of quantum devices and protocols. By focusing on direct measurement of key properties rather than full state reconstruction, the fundamental principles of our approach enable the creation of more resource-efficient quantum algorithms. Moreover, combining a classical neural network with quantum measurements demonstrates the power of hybrid quantum-classical computing. In summary, our resource-efficient approach to measuring quantum correlations represents a significant step toward practical quantum characterisation techniques for quantum devices in the near future. We respond to the growing demand for efficient entanglement verification methods on currently available quantum hardware.

## Supplementary Information

Supplementary Information.

## Data Availability

All relevant data and code supporting the document is available upon request. Please refer to Patrycja Tulewicz at patrycja.tulewicz@amu.edu.pl.
