# Supplementary material for: Resource-efficient quantum correlation measurements via multicopy neural network methods
Source: Sci Rep. 2025 Nov 19;15:40868. doi: 10.1038/s41598-025-24607-2 (PMC12630988; doi:10.1038/s41598-025-24607-2)
Supplement: Supplementary file 1 — Supplementary Information. [file 41598_2025_24607_MOESM1_ESM.pdf]

Appendices

Quantum Circuit Implementation

Accurate construction and manipulation of quantum circuits is key to the successful implementation of our multicopy measurement approach. By carefully manipulating multiple copies of the quantum state, we have created customized circuits that exactly reproduce Hong-Ou-Mandel interference.

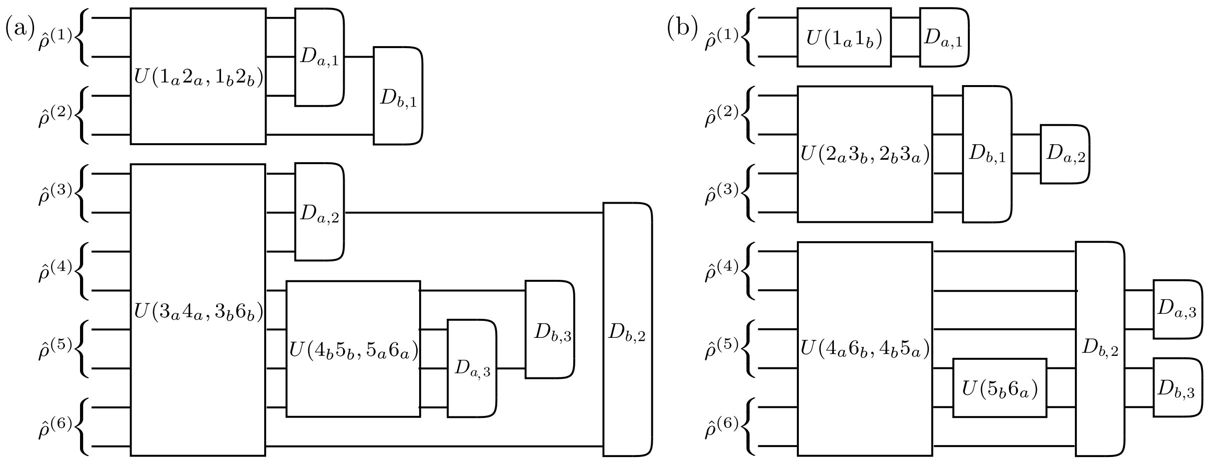

Figure S1. Implementation of singlet projection measurements. (a) Circuit configuration for measuring l_1, l_2, c_1, c_2, c_3, $\bar{c}_3$, c_4, and c_5. (b) Circuit configuration for measuring l_0, $\bar{c}_1$, $\bar{c}_2$, $\bar{l}_1$, and $\bar{l}_2$. Gates $U(k_a k_b)$ and $U(k_a l_b | l_a k_b)$ are detailed in Fig. S2. Detector pairs $D_{a,n}$ and $D_{b,n}$ ($n = 1,2,3$) measure coalescence and anticoalescence, with total counts $s = a + c$ proportional to the incident pair rate.

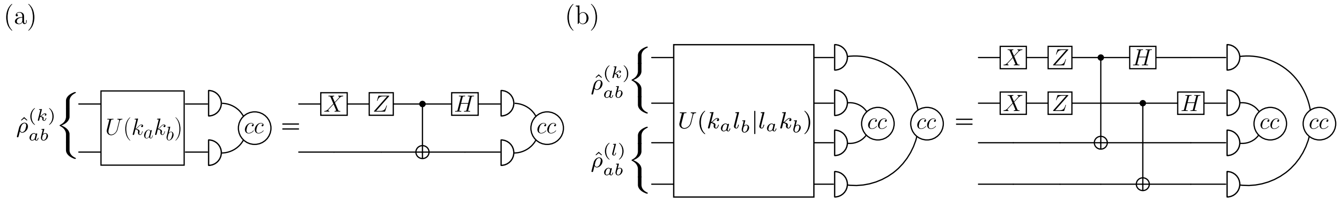

Figure S2. Fundamental circuit blocks for singlet projections. (a) Gate $U(k_a k_b)$ operation acting on the kth entangled qubit pair. (b) Gate $U(k_a l_b | l_a k_b)$ acting on two copies of qubit pairs from subsystems a and b. These blocks form the building elements for all projection measurements.

State–copy Preparation Protocol

Another important element of our approach is the simultaneous preparation of a large number of identical copies of the quantum state. We have achieved this in practice through a systematic averaging and qubit mapping process:

$$\hat{\rho}_{\text{final}} = \sum_{i=1}^{N_{\text{maps}}} w_i \hat{\rho}_i, \tag{1}$$

where w_i represents optimized weights determined by hardware characteristics

$$w_i = \frac{\exp(-\sum_j \varepsilon_{ij})}{\sum_k \exp(-\sum_j \varepsilon_{kj})}, \tag{2}$$

where, ε_{ij} represents the error rate for the jth operation in mapping i. This averaging method significantly improves the fidelity of our multicopy measurements.

Measurement Configurations

Table S1 illustrates various detector settings employed to measure different correlation terms in Fig. S1. The rows correspond to various combinations of coalescence (a) and summed signal (s) over detector pairs $D_{a,n}$ and $D_{b,n}$. By projecting these results onto l_i and c_i, or their barred counterparts, we can efficiently acquire properties such as singlet projections and correlation coefficients. This systematic labeling supports repeatability and transparency in interpreting the measurement results.

Table S1. Detector configurations and their corresponding measurements for the circuits in Fig. S1. Here a represents anticoalescence, s represents the sum signal.

$D_{a,1}$	$D_{a,2}$	$D_{a,3}$	$D_{b,1}$	$D_{b,2}$	$D_{b,3}$	(a)	(b)
a	s	s	a	s	s	l_1	–
s	a	a	s	a	a	l_2	–
s	s	s	s	s	a	c_1	–
s	s	a	s	s	s	c_2	–
s	a	s	s	a	s	c_3	–
s	s	a	s	a	a	$\bar{c}_3$	–
s	s	s	a	a	a	c_4	–
s	a	s	s	a	a	c_5	–
a	s	s	s	s	s	–	l_0
s	s	s	a	s	s	–	$\bar{c}_1$
s	s	s	s	a	a	–	$\bar{c}_2$
s	a	s	a	s	s	–	$\bar{l}_1$
s	s	a	s	a	a	–	$\bar{l}_2$

Shot Noise Dependencies

Figure S3 shows the standard deviations of the negativity N and the nonlocality measure B as a function of the number of shots. Panels (a,b) compare QST results for the Werner and Horodecki states, respectively, and (c,d) compare the corresponding for MCE. The higher the number of shots, the lower the statistical fluctuations; however the rate of this improvement depends on the measurement protocol. These plots show that MCE can potentially achieve similar or even improved accuracy with fewer shots under certain regimes because it was a smaller set of more informative measurements.

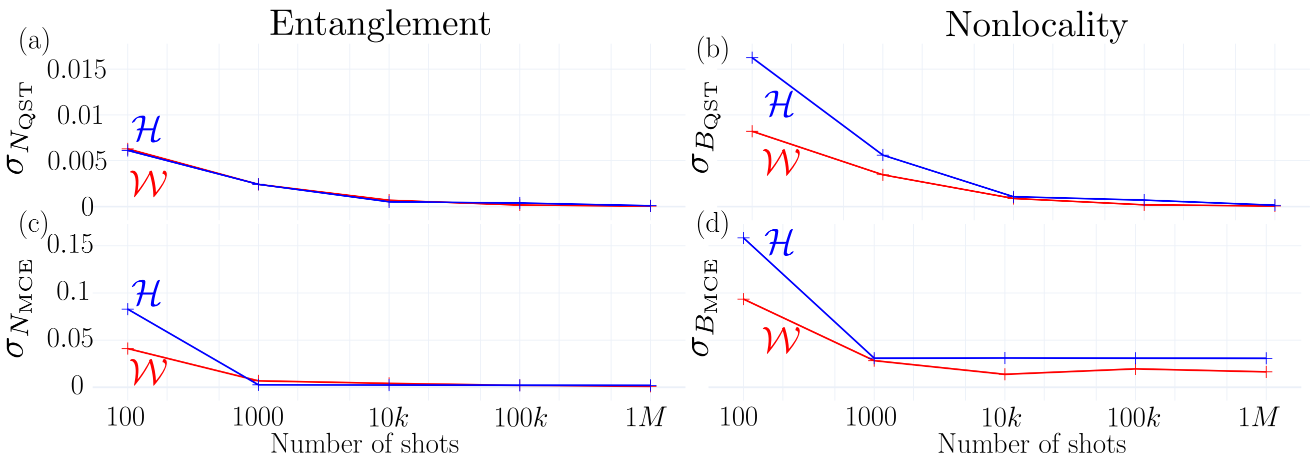

Figure S3. Measurement precision analysis. Standard deviations of (a,c) the negativity N and (b,d) nonlocality parameter B measurements as functions of shot count. Results shown for both (a,b) QST and (c,d) MCE methods, applied to the Werner (W) and Horodecki (H) states.

Hardware Characteristics

Finally, Fig. S4 provides calibration data for all qubits of the *ibm_hanoi* processor used in our experiment. We show one- and two-qubit gate error histograms and readout infidelities for the subset of qubits $\{1-5, 7, 10, 12-14, 16, 19\}$. The hardware features must be interpreted and adapted so that they can be addressed and optimized using multicopy measurement circuits to achieve the highest possible fidelity.

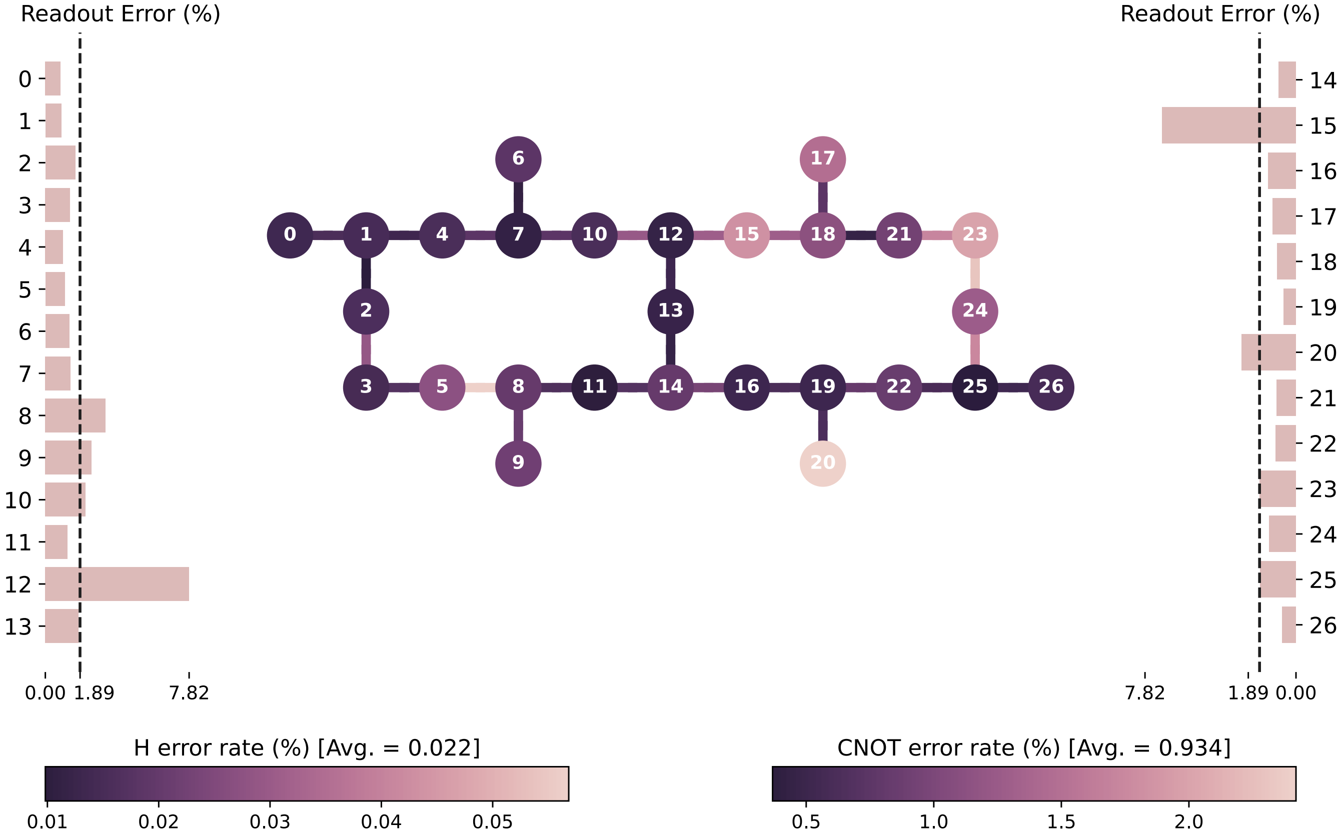

Figure S4. Error characterization of the *ibm_hanoi* processor. Calibration data shown for the relevant qubit subset $\{1-5, 7, 10, 12-14, 16, 19\}$ used in our experiments.
